# Feasibility and acceptability study of an engagement intervention for family members in early intervention programs for psychosis

**DOI:** 10.1038/s41537-025-00701-2

**Published:** 2025-12-03

**Authors:** Oladunni Oluwoye, Bryony Stokes, Karina Silva Garcia, Michael T. Compton, Dennis G. Dyck, Roberto Lewis-Fernández, Sterling M. McPherson, Leopoldo J. Cabassa, Michael G. McDonell

**Affiliations:** 1 Department of Community and Behavioral Health, Elson S. Floyd College of Medicine, Washington State University, Spokane, WA USA; 2 Department of Psychiatry, Columbia University and the New York State Psychiatric Institute, New York, NY USA; 3 Department of Psychology, Washington State University, Pullman, WA USA; 4 George Warren School of Social Work, Washington University in St. Louis, St. Louis, MO USA

**Keywords:** Psychosis, Schizophrenia

## Abstract

Despite evidence supporting the involvement of family members in early-intervention services for psychosis, rates of family engagement have been relatively low, and disparities exist. This study investigated the acceptability, feasibility, and preliminary impact of Family Motivational Engagement Strategy (FAMES) with family members of clients enrolled in coordinated specialty care (CSC). A feasibility and acceptability pilot study of FAMES was conducted in five CSC programs for FEP using a modified stepped-wedge design. FAMES consists of brief weekly contacts based on communication preferences (i.e., phone, text messages, email) and the use of culturally responsive strategies over 12 weeks. Assessments were completed at baseline and weeks 4, 8, and 12. Primary outcomes were feasibility and acceptability, and secondary outcomes were engagement in FAMES and CSC. Forty-three participants were recruited (approximately 85% of the target recruitment sample of 50) and 72% (*n* = 31) completed all 12 weeks. Participants reported high rates of satisfaction with FAMES. Regarding engagement, 86% of scheduled FAMES appointments were attended, and no significant ethnoracial differences in engagement were observed. Exploratory analyses revealed engagement in FAMES was associated with engagement in CSC. Findings demonstrated the feasibility and acceptability of delivering FAMES within CSC settings for family members/support persons. A subsequent study is needed to examine the efficacy and real-world implementation of FAMES.

## Introduction

Coordinated specialty care (CSC) and other early-intervention models are considered the gold standard of care for first-episode psychosis (FEP)^1^. CSC is comprised of evidence-based treatments and practices, including family education and support (e.g., family psychoeducation)^2–7^. In the development of CSC models in the U.S., family education and support was included as a core component due to the vital role family members often play in providing emotional and practical support for many individuals with FEP. For example, family members assist with navigating and initiating mental health services, scheduling appointments, advocacy, and medication adherence^8^. Providing education and support services to family members also helps them directly, improving their ability to care for individuals with FEP^2,5,9^. Benefits to family members include improved coping skills, increased knowledge and skill building, and reduced burden, distress, and expressed emotion^10^.

The benefit of engaging family members in the care of their loved one leads to positive impacts on clinical and functional outcomes for those enrolled in services. Research has shown that family involvement increases client engagement, decreases relapse, and improves quality of life^11–14^. Given the role family members play across the continuum of care for psychosis, their engagement in their loved ones’ care is vital. Yet, studies have shown low rates of family engagement in CSC despite the benefits previously identified, and some studies have revealed ethnoracial disparities.

Using data from the Recovery After an Initial Schizophrenia Episode—Early Treatment Program (RAISE-ETP), Oluwoye and colleagues found that fewer than 50% of participants’ family members attended family education and support appointments across two years^15^. Observational studies using data collected from CSC implemented throughout the U.S. report not-dissimilar findings. In Illinois and Minnesota, approximately 55% of clients enrolled in CSC had family members who attended at least one appointment in the first year of CSC^12,16^. In Washington State’s network of CSC services, 40% of clients had family members who attended appointments in the first month; family engagement was higher over the subsequent two years, with 70% attending 1+ appointments^17^. Additionally, family engagement earlier in the process of CSC led to longer duration of engagement among clients enrolled in services^11^. Ethnoracial disparities are apparent in family engagement in CSC, with ethnoracially minoritized families engaging in services at even lower rates than non-Hispanic white families^12,15,18^. These findings evidence the need to improve family engagement in many CSC programs throughout the U.S., with specific attention to reaching ethnoracially minoritized family members and those living in underserved areas.

Qualitative studies have identified individual and logistical factors as possible explanations for low rates of family engagement, including lack of perceived relevance, cultural misalignment, transportation barriers, and work conflicts^19,20^. However, these factors are not the only reasons family member may not engage in mental health services. Program-level factors such as limited office hours can also hinder engagement^19^. Several strategies and approaches have been found to address some of these barriers to engagement in mental health services. For instance, evidence suggests the Cultural Formulation Interview (CFI) builds trust, improves communication, and facilitates cultural responsiveness^21–23^. To mitigate geographical and logistical constraints, phone-based interventions (e.g., text messaging) offer flexible and convenient communication channels to promote family engagement^20,24^. As such, potential interventions should strive to mitigate known barriers to engagement reported by family members, as well as incorporate strategies to bridge engagement disparities.

We are aware of no studies investigating engagement interventions for family members in CSC programs or other early-intervention settings for FEP. To address this gap, the present paper describes the pilot trial of a brief engagement intervention, Family Motivational Engagement Strategy (FAMES), developed to improve family engagement in the family education and support component of CSC. Specifically, we sought to examine the feasibility and acceptability of FAMES among family members of individuals in the early stages of psychosis enrolled in CSC.

## Results

### Demographic characteristics of participants

Forty-three family members participated in the study; their sociodemographic characteristics are presented in Table 1. The mean age of participants was 49.57 years (*SD* = 10.11), and most participants identified as female and a parent of a loved one enrolled in CSC (*n* = 35; 81%). Regarding race and ethnicity, the majority of participants identified as an ethnoracial minority, with 35% (*n* = 15) identifying as Hispanic, followed by Black/African American (*n* = 5; 11.6%). Roughly 16% (*n* = 7) of participants recruited were monolingual Spanish-speakers, with remain participants English or bilingual speakers. On average, participants had a loved one enrolled in CSC for approximately two months (*M* = 2.08, *SD* = 1.54) at the start of FAMES.

**Table 1:** Summary of participant characteristics (*N* = 43).

Demographics	*N* (%)
Age (years)—mean (SD)	49.57 (10.11)
Months receiving CSC services—mean (SD)	2.08 (1.54)
Gender identity	
Male	7 (16.3%)
Female	35 (81.4%)
Non-binary	1 (2.3%)
Ethnoracial identity	
Non-Hispanic White	19 (44.1%)
Hispanic	15 (34.9%)
Black/African American	5 (11.6%)
American Indian/Alaska Native	1 (2.3%)
Asian	2 (4.7%)
Multi-racial	1 (2.3%)
Sexual orientation	
LQBTQ+	3 (7.0%)
Relationship to individual receiving CSC	
Parent	35 (81.4%)
Partner	4 (9.3%)
Grandparent	2 (4.7%)
Other (Sibling/Aunt)	2 (4.7%)
Language	
Monolingual Spanish speaking	7 (16.3%)
Employment status	
Full-time	18 (41.8%)
Part-time	10 (23.3%)
Unemployed	7 (16.3%)
Other^a^	8 (18.6%)
Socioeconomic status	
At or below poverty level based on household size	7 (16.3%)
Preferred method of contact	
Phone	35 (81.4%)
Text	3 (7.0%)
Email	5 (11.6%)

^a^Includes keeping house, retired, and temporary leave.

### Feasibility

A total of 51 family members of CSC-enrolled clients completed study interest forms, and 43 (84%) participants were assessed for study eligibility. All were consented and enrolled in the study. Eighty-six percent of the original target sample size (*N* = 50) was recruited. Of the eight family members who initially expressed interest in participating but were not enrolled in the study, one declined to participate because they were content with the number of services received at the time, two declined due to time constraints, and three could not be reached. Thirty-one participants (72%) completed the trial (i.e., 12 weeks). Twelve (28%) participants discontinued the study prematurely (6 [14%] during weeks 10 and 11 and 6 [14%] during weeks 2 to 9) for the following reasons: moved to another geographic region, extended vacations, change in job hours, decreased desire to participate, and unknown reasons. No adverse events occurred during the trial period.

### Acceptability

**Fig. 1:**
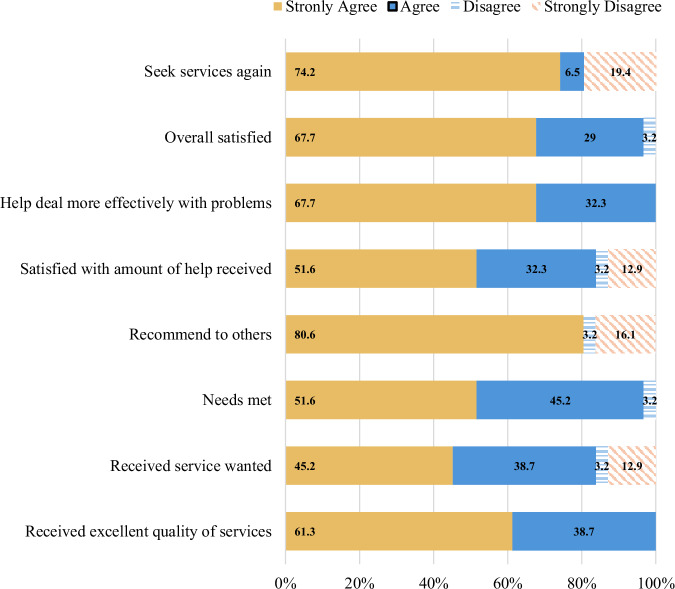
Participant satisfaction ratings on the client satisfaction questionnaire. The percentage distribution of participant’s responses to each item on the Client Satisfaction Questionnaire – 8 (*n* = 30).

At week 12, approximately 97% of participants (*n* = 30) reported being satisfied to very satisfied with FAMES. The average total score on the CSQ-8 was 27.61 (*SD* = 4.88). Participant satisfaction ratings for each item on CSQ-8 are displayed in Fig. 1.

### Engagement

Overall, the mean duration spent in FAMES was 10.84 weeks (*SD* = 2.68; range 2–12 weeks). A total of 495 FAMES appointments were scheduled, and 86% of the scheduled appointments were attended. The mean length of time for appointments was 21.58 min (*SD* = 15.47) and ranged from 4 to 80 min. Appointments during the Building Phase lasted approximately 21.26 min (*SD* = 15.64) and Continuous Contact Phase appointments lasted approximately 21.73 min (*SD* = 15.41). Seventy-three percent (*n* = 379) of outreach efforts were successful on the first attempt. As shown in Table 2, engagement in FAMES slightly varied by ethnoracial group; however, post-hoc analyses revealed no significant ethnoracial differences in the number of weeks participants engaged in FAMES (*F* [3, 39] = 0.68, *p* = 0.573) and the number of scheduled appointments attended (*F* [3, 39] = 1.66, *p* = 0.194).

**Table 2:** Engagement in FAMES by ethnoracial group (*N* = 43).

Engagement indicators (mean/SD)	Ethnoracial group				*p* value
	Non-hispanic white (*n* = 19)	Hispanic (*n* = 15)	Black/African American (*n* = 5)	Other (*n* = 4)	
Duration of engagement (weeks)	11.21 (2.13)	10.67 (3.15)	9.40 (3.97)	11.25 (0.96)	0.573
Number of appointments attended	10.84 (2.36)	8.73 (3.69)	8.00 (3.46)	10.75 (0.96)	0.194
Phone call duration (minutes)	20.55 (10.78)	25.46 (13.78)	20.90 (7.88)	23.00 (11.61)	
Text messages per week	1 (-)	2.21 (1.40)	5.67 (8.14)	-	
Email per week	1.27 (0.29)	-	-	-	

Exploratory analysis indicated that the main effect of our linear regression, total proportion of scheduled FAMES appointments attended, was positively associated with attending family psychoeducation appointments (*β* = 0.36, *p* = 0.043). Findings suggest that greater FAMES engagement was associated with increased family psychoeducation attendance.

## Discussion

We conducted a pilot study of FAMES, an engagement intervention, in CSC programs to examine its feasibility and acceptability. FAMES was found to be feasible based on the number of participants recruited relative to the target sample size (86%) and study completion rate (72%), which have been used in prior studies as benchmarks for feasibility (e.g., ≥ 70% study completion rate)^25–27^. Family member participants reported high levels of satisfaction with FAMES. We also observed high rates of engagement in FAMES (86% of appointments attended) and did not find significant ethnoracial differences in the number of appointments attended or duration of engagement. Exploratory analyses indicated there was an association between engagement in FAMES and engagement in the family psychoeducation component of CSC within the first six months of services. Taken together, these findings provide initial support for FAMES’s potential to improve family engagement in CSC.

Overall satisfaction with FAMES was high, with all but one participant reporting being satisfied and very satisfied. Family members’ highest satisfaction ratings were with the quality of services, needs being met, and helpfulness of the approach. Despite all participants rating the quality of services as excellent, approximately four participants were indifferent or dissatisfied with the amount of help received. This may be due to the frequency (i.e., weekly) in comparison to contact made monthly through family psychoeducation appointments and the 12-week duration of FAMES, which could be demanding and an added stressor for some families, even in its brevity. Also, the average duration of engagement among participants was between 10 and 11 weeks, potentially indicating that families may still benefit from a shorter duration for FAMES. It is possible that the modifications to the delivery of FAMES, which included having bilingual Spanish-speaking staff and individuals with lived experience of a family member delivering FAMES, may have contributed to the satisfaction ratings and engagement among participants. Research has shown that alignment of cultural factors such as language and ethnoracial identity contributes to rapport building and satisfaction in mental health services^28,29^. In addition, family peer roles, while not common in CSC programs, represent a distinct role that differs from the clinical roles on a team, often serving as a bridge between families and clinicians^30^. These modifications may offer insight on individualized approaches that could facilitate family engagement in CSC.

The potential flexibility encompassed within FAMES, such as using client communication preferences (83% preferred phone calls), offering unconventional hours to connect with family members, and the use a modified CFI for families may address known barriers to care identified in prior research^19^. While CFI is known for improving rapport and communication^22^, our unique modifications to the CFI may have further facilitated the understanding of previous barriers, current needs, and areas of greatest importance to family members (e.g., type of support or resources, integration of cultural values). The first four appointments of FAMES (i.e., Building Phase), which is structured and informed by the CFI, are similar to work conducted by Aggarwal and colleagues, who tested the implementation of CFI-Engagement Aid (CFI-EA) in community a community hospital to improve treatment adherence and engagement across three appointments^31^. However, unlike the CFI-EA, FAMES also includes a Continuous Contact Phase that integrates cultural and contextual factors salient to family members and focuses on discussing and addressing factors related to motivation towards engaging in the family education and support component of CSC. While the CFI has not traditionally focused on the family members of individuals enrolled in mental health services, FAMES was developed with the specific intention of centering family members and improving their engagement in care. This study contributes to the paucity of CFI-related studies that include family members^22,32^, and can potentially serve as guide on the integration and adoption of the CFI into larger family-based interventions.

Many CSC programs encounter challenges engaging family members early in the family education and support component of CSC^12,16–18^. Given that prior research has focused on the importance of engaging family members within the first few months of CSC because of its association with sustained engagement^11^, the present study prioritized engaging family member participants early in care. On average, we identified and connected family member participants with FAMES in the first two months of CSC. Related to our secondary outcomes, this study prioritized recruiting an ethnoracially diverse sample of participants and while engagement in FAMES varied across ethnoracial groups, ad-hoc analyses revealed no significant differences. Our exploratory results revealed an association between greater family members’ engagement in FAMES and higher attendance at CSC appointments. These findings suggest that FAMES may be the behavioral nudge needed to facilitate engagement in existing services/components of CSC, thus increasing family engagement in CSC.

### Limitations

There are several limitations that should be considered when interpreting our findings. This study’s main purpose was to examine feasibility and acceptability; due to sample sizes associated with feasibility studies, our study was not powered to conduct statistical analyses of potential efficacy. Our stepped-wedge pilot study lacked randomization and a control group to explore the direct impact of intervention outcomes. Recruitment commenced during the height of COVID, and the initial study design and recruitment strategies, outlined in a published protocol paper^33^, were modified to accommodate changes at CSC sites. Although the gender and role composition (i.e., majority female and parents) was relatively consistent with other family-based studies^34–36^, few males, fathers, and siblings were recruited and have generally been underrepresented in family-based interventions^37,38^. Due to the critical relational role and support that fathers and siblings play^39^, future studies may want to prioritize the recruitment of multiple family members to understanding unique and congruent experiences and various types of family and supportive roles for individuals in the early stages of psychosis. While our study included five CSC programs at various behavioral health agencies in geographically diverse locations (i.e., urban, rural), sites were all part of a large network of CSC services in Washington State. Findings may not be generalizable to CSC networks in other states that use alternative CSC models and systems of care. CSC models differ across states, including team structures and workflows. Future research will need to assess implementation of FAMES in various CSC models, such as NAVIGATE-based, OnTrack, or EASA.

## Conclusions

Findings highlight the potential of FAMES to address family engagement in CSC settings and actively encourage engagement among ethnoracially diverse and underserved families in CSC. Given the expansion of CSC throughout the U.S., family-based interventions that can be integrated into such models to facilitate engagement are needed. This study represents the first step, and further research is warranted that should include a larger randomized effectiveness trial of FAMES that also considers implementation needs to better understand implications for the real-world roll-out in CSC settings.

## Methods

### Study design

This pilot study was conducted with five CSC programs in community behavioral health settings using a non-randomized modified stepped-wedge design. Each CSC program represented a cluster, individually allocated to a step using a convenience-assignment approach. A 29-month open-cohort design was used, with each CSC program allotted a 12-month intervention phase to recruit participants. Modifications to the initial study design (e.g., extended recruitment timelines) outlined in the study protocol were made due to changes in the delivery of behavioral health care during COVID-19 social distancing mandates^33^. This pilot trial occurred from April 2021 through August 2023 and was pre-registered with clinicaltrials.gov (NCT04188366).

### Participants

Consistent with prior feasibility pilot studies in clinical settings, the target sample size for the present study was *N* = 50. Inclusion criteria for family member participants were: (1) aged 18 years or older; (2) being a family member (parent, sibling, etc.) of an individual in the early stages of psychosis enrolled in CSC for less than six months; and (3) ability to speak and understand English or Spanish. Exclusion criteria included participation in the intervention development phases of FAMES described elsewhere^33^.

### Family motivational engagement strategy (FAMES) intervention

FAMES was delivered over 12 weeks and consisted of brief weekly contact divided into two phases: (1) Building and (2) Continuous Contact, according to the family member’s preferred methods of communication (i.e., phone, text message, or email). Based on feedback obtained during a six-month open trial with five family members and due to the impact of COVID-19 on CSC program workflow, modifications were made to the original FAMES approach described in the initial protocol^33^. For example, while the original plan was to train program directors and family education/support providers to deliver FAMES, workflow changes led to its delivery by two research coordinators—one with lived experience as a family member of someone with serious mental illness, and one bilingual in English and Spanish—who worked with families of individuals enrolled in CSC.

The Building Phase consisted of four appointments. The first two appointments integrated a modified version of the CFI consisting of five domains (defining the problem; stressors and supports; role of cultural identity; cultural factors affecting coping and past help seeking; and cultural factors affecting current help seeking)^40^. Family members were asked two to five questions within each domain to identify cultural and contextual factors (Supplementary Table S1). On a scale of 1 (not important) to 10 (high importance) family members were asked to rate the following: (1) addressing challenges with their loved ones, (2) finding ways to manage stressors, (3) integrating cultural identity into FAMES sessions, (4) developing coping skills specific to culture, and (5) feeling included in loved ones CSC services. The following two sessions involved discussion to further understand the nature of relationships with their loved ones and others, and caregiving activities. The information gathered from the CFI was used to set goals and expectations for future FAMES sessions.

The Continuous Contact Phase included eight semi-structured check-ins inquired about key areas (social context, clinical support), allow participants to raise topics most relevant to them (e.g., challenges and successes throughout the week), and discuss motivational factors, all while integrating cultural and contextual factors identified during the Building Phase (Supplementary Table S2). At the end of each check-in, motivational factors were discussed, and family members were asked to indicate their level of motivation towards participating in monthly CSC on a scale of 0 (unmotivated) to 10 (highly motivated). Based on family members’ ratings follow up discussion occurred to understand factors that drive motivation to participate, factors that could result in losing motivation, and to identify weekly goals. Throughout both phases, family members were reminded about upcoming family education and support appointments scheduled with their CSC. Information obtained during the Building and Continuous Contact Phases was shared with family education and support providers at each CSC site to address immediate queries or concerns by email and to improve the relevance of family education and support sessions.

### Study procedures

Study staff provided each CSC program with study interest forms and flyers to distribute to enrolled clients and their families. Program directors emailed all completed study interest forms to study staff. Potential participants were contacted and informed about the study purpose, procedures, and potential risks and benefits. If interested, potential participants were screened for eligibility, and informed consent was obtained from all eligible participants using Research Electronic Data Capture (REDCap) e-consenting procedures. Refusal to participate in the study was documented by study staff. All study materials were translated and back-translated into Spanish, and a bilingual study staff member conducted all weekly appointments with monolingual Spanish-speaking participants. Assessments were captured in REDCap at baseline and monthly. For completing baseline and monthly assessments, participants were compensated $20 and $10, respectively. Washington State University’s Institutional Review Board approved all study procedures.

### Measurements

#### Feasibility outcomes

Consistent with CONSORT guidelines for pilot/feasibility studies, feasibility outcomes included the following: consent rates, total number of participants recruited, retention rates, study completion rates, dropout rate, and number of adverse events^41^. Consistent with prior studies^25^, 75% recruitment of the target sample size and 70% study completion rate were set as the benchmarks for feasibility.

#### Acceptability outcomes

The Client Satisfaction Questionnaire-8 (CSQ-8) is a brief self-report measure used to assess satisfaction and quality, which has good psychometric properties^42^. The CSQ-8 has been extensively studied and used to examine acceptability in mental health settings^43^. This 8-item measure is rated on a 4-point Likert scale, with total scores ranging from 8 to 32. Higher scores on the CSQ-8 indicate greater satisfaction; scores > 23 have been suggested as the benchmark for acceptability^44,45^.

#### Engagement outcomes

Engagement in FAMES was assessed weekly using the number of scheduled FAMES appointments attended by phone, email, or text. To assess engagement among participants whose communication preferences were text or email, the initial participant response to weekly text messages and emails was rated as attended. Engagement in family psychoeducation appointments was captured from affiliated CSC programs as the proportion of scheduled appointments attended each month during the 12-week period participants were enrolled in the study.

### Statistical analysis

Preliminary data analyses were primarily descriptive due to the relatively small sample size. To assess acceptability and feasibility, descriptive statistics (e.g., means, standard deviations, frequencies, percentages) were used to summarize primary (feasibility and acceptability) and secondary (engagement in FAMES) outcomes. A follow-up analysis of covariance (ANCOVA) was conducted to assess whether there were ethnoracial differences in the duration of engagement and number of FAMES appointments attended, with age and site included as covariates. Using linear regression, exploratory analyses were conducted to examine whether the proportion of scheduled FAMES appointments attended by participants over the course of 12 weeks predicted the proportion of scheduled CSC services attended during the concurrent period, controlling for age and CSC site. Analyses were performed in SPSS and all tests were two-sided with a *p* < 0.05.

## Supplementary information

Supplemental Tables

## Data Availability

The data that supports the findings of this study will be openly available from the corresponding author upon reasonable request at the time of publication.
