# Supplementary material for: Feasibility and acceptability study of an engagement intervention for family members in early intervention programs for psychosis
Source: Schizophrenia (Heidelb). 2025 Dec 3;12(1):5. doi: 10.1038/s41537-025-00701-2 (PMC12775528; doi:10.1038/s41537-025-00701-2)
Supplement: Supplementary file 1 — Supplemental Tables [file 41537_2025_701_MOESM1_ESM.docx]

**SUPPLEMENTARY TABLES**

| **Table S1.** Building Phase: Structured appointment questions informed by the Cultural Formulation Interview |
| --- |
| **Defining the Problem** **– Appointment 1** |
| Sometimes people have different ways of describing their problems to their family, friends, or others in their community. How would you describe your experience with your loved one? |
| What troubles you most about having a loved one [experiencing psychosis/diagnosis]? |
| On a scale of 1-10, how important is it for you to find ways to manage the stressors you are experiencing? |
| **Stressors and Supports – Appointment 1** |
| Are there any kinds of support that make your experience having a loved one experiencing first episode psychosis better, such as support from family, friends or others? |
| Are there any kinds of stresses that make your experience having a loved one experiencing first episode psychosis worse, such as difficulties with money, or family problems? |
| On a scale of 1-10, how important is it for you to find ways to manage the stressors you are experiencing? |
| **Role of Cultural Identify – Appointment 1** |
| For you, what are the most important aspects of your background or identity? |
| Are there aspects of your background or identify that make a difference to your experiences having a loved one [experiencing psychosis/diagnosis]? |
| Are there any aspects of your background or identity that are causing other concerns or difficulties for you? |
| On a scale of 1-10, how important is it for you to have your cultural identity incorporated into your FAMES sessions? |
| **Cultural Factors Affecting Self Coping and Past Help Seeking – Appointment 2** |
| Sometimes people have various ways of dealing with problems, concerns, and coping. What have you done on your own to cope with your experience of having a loved one [experiencing psychosis/diagnosis]? |
| Often, people look for help from many different sources, including different kinds of doctors, helpers, or healers. In the past, what kinds of treatment, help, advice, or healing did you seek for your experiences having a loved one [experiencing psychosis/diagnosis]? |
| What types of help or treatment were most useful and not useful? |
| Has anything prevented you from getting the help you need? |
| On a scale of 1-10, how important is it for you to develop coping skills specific to your culture? |
| **Cultural Factors Affecting Current Help Seeking – Appointment 2** |
| What kind of help do you think would be most useful to support you at this time? |
| Are there other kinds of help that your family, friends, or other people have suggested would be helpful for you now? |
| Sometimes there are misunderstandings because people come from different backgrounds or have different expectations. Have you been concerned about this, and is there anything that we can do to provide you with the care you need for yourself? |
| On a scale of 1-10, how important is it for you to feel included in your loved ones’ CSC care and treatment planning? |
| **Nature of Relationship** **– Appointment 3** |
| How are you connected to the individual receiving care? |
| How long have you been taking care of the individual receiving care? |
| How, if at all, has your relationship changed since their first episode of psychosis? |
| **Caregiving Activities – Appointment 4** |
| How do you help your loved ones on a day-to-day basis? |
| What is the most rewarding and challenging things about helping or supporting them? |
| Are there any cultural traditions that influence how you approach helping or supporting your loved one? |
| Is the amount or kind of help you are giving different in any way from what would be expected in the community that you are from? |

| **Table S2.** Continuous Contact Phase: Semi-structured check-in appointment questions |
| --- |
| **Unstructured Discussion – Appointments 5 to 12** |
| How have you been since we last spoke? |
| **Optional Prompts for Inquire Areas – Appointments 5 to 12** |
| How are you coping? |
| Are there other, such as family members, friends, or neighbors, who also help you or your loved one? |
| How do you feel about how much or how little others are helping you during this time? |
| How can we make it easier for you to be able to help your loved one? |
| **Motivational Statements – Appointments 5 to 12** |
| On a scale of 1-10, how motivated do you feel to participate in family education and support appointments? |
| What drives you to want to take part in services related to your loved one’s care? |
| Are there any factors that could result in you losing motivation to continue taking part in services related to your loved ones’ care? |
| Do you have any goals for: 1. yourself this week? 2. Your next FAMES appointment; and 3. family education and support appointment, that you need or want assistance with? |
